# Expression Levels of DNMT1, HDAC1, and MT1E Genes as Diagnostic Biomarkers for Breast Cancer Clinicopathological Features: In Vitro and In Silico Analyses

**DOI:** 10.1155/ancp/9606190

**Published:** 2026-07-21

**Authors:** Sedigheh Akhtartavan, Hamid Madanchi, Raheb Ghorbani, Abolfazl Khalafi-Nezhad, Ahmad Abdullahi, Fahimeh Shamsi, Hossein Heli

**Affiliations:** ^1^ Department of Medical Biotechnology, Semnan University of Medical Sciences, Semnan, Iran, semums.ac.ir; ^2^ Department of Epidemiology and Biostatistics, Semnan University of Medical Sciences, Semnan, Iran, semums.ac.ir; ^3^ Department of Hematology, Shiraz University of Medical Sciences, Shiraz, Iran, sums.ac.ir; ^4^ Cellular and Molecular Research Center, Gerash University of Medical Sciences, Gerash, Iran, en.gerums.ac.ir; ^5^ Nanomedicine and Nanobiology Research Center, Shiraz University of Medical Sciences, Shiraz, Iran, sums.ac.ir

**Keywords:** diagnostic biomarkers, DNMT1, epigenetic regulators, HDAC1, MT1E, triple-negative breast cancer

## Abstract

**Background:**

Breast cancer (BC) is the second leading cause of cancer‐related deaths worldwide and has a high recurrence rate. This study aimed to evaluate the expression levels of three biomarkers: DNA methyltransferase 1 (DNMT1), histone deacetylase 1 (HDAC1), and metallothionein 1E (MT1E) in BC patients.

**Methods:**

Peripheral blood and tissue samples from 95 female BC patients and 50 age‐matched (±5 years) healthy female controls were analyzed using an enzyme‐linked immunosorbent assay (ELISA). Diagnostic potential was assessed through receiver operating characteristic (ROC) curve analysis. In silico analyses identified differentially expressed genes (DEGs), Gene Ontology (GO) terms, pathway enrichment, correlation analysis, miRNA–mRNA interactions, and drug‐gene networks.

**Results:**

Both experimental and computational results revealed significantly higher levels of DNMT1 and HDAC1 but lower levels of MT1E in BC patients compared to controls (*p*‐value < 0.0001). Furthermore, tumor expression of these genes correlated with molecular subtypes, showing distinct expression patterns in triple‐negative BC (TNBC). Advanced‐stage tumors also exhibited increased HDAC1 and DNMT1 expression. GO enrichment analysis indicated that these DEGs are involved in cell division, differentiation, and nuclear functions. Kyoto Encyclopedia of Genes and Genomes (KEGG) pathway analysis linked DNMT1 to the p53 pathway, HDAC1 to mismatch repair, and MT1E to cytokine receptor interactions. The strongest gene correlations were DNMT1‐ILF3, HDAC1‐RBBP4, and MT1E‐MT2A. Additionally, miRNA–mRNA interaction analysis revealed that DNMT1, HDAC1, and MT1E are targeted by multiple miRNAs, with the top interacting miRNAs being hsa‐miR‐103a‐3p for DNMT1, hsa‐miR‐34a‐5p for HDAC1, and hsa‐miR‐126‐3p for MT1E. DNMT1 showed the highest number of miRNA interactions among the three genes. Moreover, 98 drugs were found to interact with these three genes. ROC analysis demonstrated promising diagnostic performance, with areas under the curve (AUC) of 0.916 for DNMT1, 0.792 for HDAC1, and 0.683 for MT1E (95% confidence interval [CI]).

**Conclusion:**

These findings suggest that DNMT1, HDAC1, and MT1E show potential as complementary biomarkers in BC diagnosis. However, further validation in larger cohorts and functional studies are warranted.

**Summary**

• Higher DNA methyltransferase 1 (DNMT1) and histone deacetylase 1 (HDAC1) levels were measured in breast cancer (BC) patients.
• A lower metallothionein 1E (MT1E) level was measured in BC patients.
• Tumor expression of the genes showed elevated levels in triple‐negative BC (TNBC).

## 1. Introduction

The most common malignant tumor among women is breast cancer (BC), which is the second leading cause of cancer‐related deaths worldwide and has a 10%–40% recurrence rate [1]. Surgery, chemotherapy, hormone therapy, and radiotherapy are among the therapeutic approaches used at different stages of BC treatment [2]. However, despite advancements in chemotherapy over the past 3 years, current drugs remain ineffective in adequately targeting tumors. It is estimated that most cancer‐related deaths result from unsuccessful chemotherapy treatments [3]. Given the clinical and molecular limitations of individualized tumor treatment, new therapeutic strategies are needed to predict treatment outcomes and patient responses. On the other hand, early diagnosis is crucial, and identifying reliable and accessible biomarkers is essential [4] as they play a critical role in cancer treatment planning [5]. Late diagnosis significantly diminishes the effectiveness of treatment interventions. Therefore, ideal biomarkers should have clinical utilities in stratifying patients into higher‐ or lower‐risk groups, correlate well with traditional clinicopathological parameters, and predict tumor recurrence after therapy [6].

Recent studies have demonstrated that epigenetic alterations contribute to the development of many types of cancer [7]. These epigenetic modifications are considered potential tumor markers for early‐stage diagnosis. The catalysis of DNA methylation and histone deacetylation by DNA methyltransferases (DNMTs) and histone deacetylases (HDACs), respectively, plays key roles in regulating gene expression during carcinogenesis [8]. DNMTs are crucial for posttranscriptional methylation and play a role in the tumorigenesis of many cancers [9]. In mammals, DNA methylation regulates epigenetic modifications through gene silencing, X chromosome inactivation, and maintaining genome stability [10, 11]. The epigenetic regulation of the DNMT 1 (DNMT1) gene influences both cell cycle regulation and differentiation [12]. Furthermore, DNMT1 can function as an epigenetic modifier through its regulation of target genes, thereby disrupting tumor‐suppressor activity [12]. Numerous studies have confirmed that DNMT1 is significantly upregulated in various cancers [13]. Supporting this finding, Shilpi et al. [14] identified DNMTs as promising targets for developing anticancer therapeutics.

HDAC proteins are among the most important epigenetic factors in cancer treatment, particularly in BC [15]. They remove acetyl groups from acetyllysine, thereby altering the chromatin structure and regulating gene expression [16]. Studies have established a link between HDAC 1 (HDAC1) overexpression and the development of human tumors, mediated by decreased expression of tumor‐suppressor proteins [17]. Furthermore, the target specificity of HDAC1’s function makes it an ideal candidate for cancer therapy [18]. Some studies suggest that precise regulation of epigenetic modification of HDAC [19, 20] plays a crucial role in controlling tumor growth and development, making them promising targets for BC therapies. Additionally, analyses based on DNA methylation and single‐nucleotide polymorphisms have led to the identification of novel and prognostically significant genomic loci in BC [21].

Evidence has shown that metallothioneins (MTs), a superfamily of metal‐binding proteins, contribute to tumorigenesis and cancer treatment [22, 23]. MTs are single‐chain mammalian polypeptides capable of forming thiolate clusters by binding seven divalent cations to the 18–23 cysteine residues within each molecule [24]. In humans, 10 functional MT isoforms have been identified, which participate in various cellular processes that promote tumor development [25]. Using integrated bioinformatics analyses, researchers have identified multiple genetic and epigenetic variations associated with the BC diagnosis [26].

In this respect, valuable information about the origin and progression of the disease can be obtained by comparing gene expression levels in patients and healthy individuals [27]. Given the importance of biomarkers in the early diagnosis of BC, this study aims to investigate the expression levels and potential diagnostic/prognostic roles of DNMT1, HDAC1, and MT 1E (MT1E) as biomarkers in the serum and tissue of BC patients. Evaluation of clinicopathological parameters revealed that histological characteristics vary according to the molecular subtypes. Finally, the correlation between the DNMT1, HDAC1, and MT1E gene panels and clinical factors.

The novelty of this study lies in three aspects: First, unlike previous studies that examined DNMT1 and HDAC1 separately or only at the mRNA level, we simultaneously quantified all three proteins (DNMT1, HDAC1, and MT1E) in both serum and tissue samples using enzyme‐linked immunosorbent assay (ELISA), providing translational relevance. Second, our data show that within the triple‐negative BC (TNBC) subtype, despite overall downregulation of MT1E in BC, MT1E expression is significantly higher compared to luminal and HER2+ subtypes, suggesting subtype‐specific epigenetic regulation. Third, we combined experimental ELISA data with in silico miRNA–mRNA and drug–gene interaction networks specifically for this three‐gene panel, which, to our knowledge, has not been previously reported in BC.

While tissue analysis provides direct evidence of gene expression at the tumor site, serum analysis offers a noninvasive and clinically accessible approach. Comparing both compartments allows us to assess whether changes in the tumor tissue are reflected in peripheral blood, which is essential for developing blood‐based diagnostic biomarkers. However, it is important to note that serum levels may be influenced by systemic factors and do not perfectly mirror tissue expression.

## 2. Materials and Methods

### 2.1. Subject Selection

A total of 145 individual samples comprising two independent cohorts: a serum cohort (60 female BC patients and 30 age‐matched (±5 years) healthy female controls) and a tissue cohort (35 female BC patients and 20 adjacent normal tissues) were included in this study. The healthy tissue was sourced from tumor‑adjacent normal tissue of the same BC patients. The female participants ranged in age from 20 to 63 years, and sample collection was conducted in the oncology department of Amiralmomenin Gerash Hospital from August 2021 to July 2023. The serum samples and tissue samples were derived from completely separate cohorts. Therefore, the serum and tissue groups were entirely nonoverlapping. Tissue samples were obtained from BC patients undergoing surgical treatment, and the specimens were sent to a pathology laboratory for analysis. All participants provided written informed consent before enrollment.

Inclusion criteria were as follows: (1) female patients aged 20–63 years; (2) histopathologically confirmed BC; (3) no prior chemotherapy or radiotherapy; and (4) willingness to provide written informed consent.

Exclusion criteria were as follows: (1) metastatic disease outside the breast; (2) prior chemotherapy or radiotherapy; (3) history of other malignancies; (4) pregnancy or lactation; and (5) autoimmune diseases or chronic inflammatory conditions.

Patients with metastases to sites outside the breast and those who had undergone presampling chemotherapy or radiotherapy were excluded. None of the enrolled patients had received any systemic treatment (including neoadjuvant chemotherapy, radiotherapy, or targeted therapy) prior to sample collection. Treatment history was recorded from patient medical records, and only treatment‐naïve patients were included. The study achieved 94.3% power for the serum comparison and 80.0% power for the tissue comparison, as calculated using 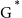
Power 3.1 based on effect sizes observed in previous studies [28, 29]. Relevant demographic and clinical history data were obtained from patient records. All samples were stored at an appropriate temperature to ensure preservation. All procedures regarding human samples were performed under ethical approval by the Semnan University of Medical Sciences (IR.SEMUMS.REC.1401.314).

### 2.2. Samples (Serum/Tissue) Processing

For serum, 5 mL of venous blood was collected from each participant using tubes (EDTA‐free). Samples were processed in less than 30 min postcollection to avoid concentration changes. For serum isolation, the sample underwent a double‐spin protocol: the initial spin was performed at 1900 × g for 10 min at 4°C to allow for initial clot formation and separation of cellular components from the liquid phase (serum). This process was then repeated (the second spin) to ensure the complete removal of any residual cells or platelets. Following clarification, 200 μL aliquots of the purified serum were immediately stored at −80°C to guarantee protein stability for downstream analyses.

For tissues, 5 mg of tissue (from snap‐freezing) was used. The tissue was lysed using RIPA buffer at a 1:10 (weight‐to‐volume) ratio. This buffer contained inhibitors, a cocktail of chemical agents designed to block the activity of protease and phosphatase enzymes, thereby ensuring that the extracted proteins remain intact. To release the intracellular proteins, the tissues were then homogenized using a TissueLyser device at a speed of 25 Hz for 2 min. Following homogenization, the resulting suspension was centrifuged at 14,000 × *g* for 15 min at 4°C to separate the soluble proteins from the insoluble cellular debris. Finally, the total extracted protein was quantified using the BCA (bicinchoninic acid) assay with the Kalazist kit (catalog number DB9684); the accuracy of these measurements was confirmed by ensuring that the coefficient of variation was less than 5%. All samples were stored at −80°C until analysis.

### 2.3. ELISA

Serum and tissue protein levels of DNMT1, HDAC1, and MT1E were quantified using commercially available human ELISA kits (DNMT1 Kit, Cat. No. E2170Hu; HDAC1 Kit, Cat. No. E2041Hu; MT1E Kit, Cat. No. E1733Hu; all from Bioassay Technology, China), following the manufacturer’s instructions. The ELISA plates were precoated with antibodies against human DNMT1, HDAC1, and MT1E. All ELISA results were normalized to total protein concentration measured by the BCA assay (for tissue) or to serum volume (for serum samples). The analytical performance of the kits was characterized according to the manufacturers’ reported precision metrics. According to the kit inserts, the intraassay CVs were <8%, and the interassay CVs were <10%. The assay range was 0.5–100 ng/mL for all three kits, with a sensitivity (detection limit) of 0.29 ng/mL. Each sample was run in duplicate, and the mean value was used for analysis.

### 2.4. In Silico Analysis

To identify the differentially expressed genes (DEGs) associated with DNMT1, HDAC1, and MT1E, we utilized the UCSC Xena platform (accessed on 15 February 2023; https://xena.ucsc.edu/cite-us) and LinkedOmics v1.6.2 (https://www.linkedomics.org/login.php). TCGA served as the primary source of cancer‐related transcriptomic data [30]. The TCGA‐BRCA dataset included 1085 BC samples and 112 normal adjacent tissue samples. Genes were considered differentially expressed when they met the criteria of |log_2_FC| > 1 and a false discovery rate (FDR) < 0.05. Volcano plots were generated to visualize the distribution of DEGs based on fold change and statistical significance. Pan‐cancer expression analysis of DNMT1, HDAC1, and MT1E was performed using UALCAN v2.0 (https://ualcan.path.uab.edu/analysis.html), with median normalization applied to compare gene expression levels between tumor and normal tissues across multiple cancer types. Gene–gene correlation analysis was conducted using the GEPIA web tool (accessed in January 2023; https://gepia.cancer-pku.cn/) to explore biological functions and pathway associations.

Functional enrichment analyses, including Gene Ontology (GO) and Kyoto Encyclopedia of Genes and Genomes (KEGG) pathways, were carried out using the DAVID v6.8 database (https://davidbioinformatics.nih.gov/tools.jsp). Pathways or terms were considered significantly enriched if the *p*‐value < 0.05 after FDR correction. The resulting GO term visualizations (GO plots) were generated using SRplot (accessed 2023; http://www.bioinformatics.com.cn/faq_en).

Experimentally validated miRNA–target interactions were obtained from miRTargetLink 2.0 (https://ccb-compute.cs.uni-saarland.de/mirtargetlink2) and ENCORI v2.0 (https://rnasysu.com/encori/contact.php). In addition, potential drug–gene interactions were investigated using the DGIdb v4.0 database (https://dgidb.org/about#faq).

### 2.5. Statistical Analysis

GraphPad Prism (8.0.1) and MedCalc statistical software (18.9.1) were used for data analysis. The normality of quantitative data was confirmed using the Kolmogorov–Smirnov test. Quantitative data were presented as the mean ± standard deviation. The Student’s *t*‐test was applied for intergroup comparisons between two cohorts. For comparisons across molecular subtypes (Luminal A, Luminal B, HER2+, and TNBC) and tumor stages, one‐way ANOVA followed by Tukey’s post hoc test was employed. For nonnormally distributed variables, the Mann–Whitney *U* test was used as an alternative. The qualitative data were presented as the number of subjects and percentage, and the chi‐square test was employed for intergroup comparisons of these variables. FDR correction using the Benjamini–Hochberg method was applied for all multiple comparisons. Furthermore, the receiver operating characteristic (ROC) curve was employed to evaluate the diagnostic performance of the expression levels of DNMT1, HDAC1, and MT1E in BC. The area under the curve (AUC) with a 95% confidence interval (CI) was calculated for each biomarker. The optimal cutoff values were selected to maximize the sum of sensitivity and specificity. Multivariate binary logistic regression analysis was performed to adjust for potential confounders including age, BMI, tumor subtype, and tumor stage, with BC status (patient vs. control) as the dependent variable. Variables with *p*  < 0.1 in univariate analysis were entered into the multivariate model. Adjusted odds ratios (ORs) and 95% CIs are reported where applicable. A *p*‐value ≤ 0.05 was considered statistically significant for all analyses.

## 3. Results

### 3.1. Demographic Characteristics

The mean ± standard deviation of the age and body mass index among the participants were 44.6 ± 12.3 years (in a range of 20–63 years) and 26.5 ± 6.2 kg/m^2^ (in a range of 14.3–42.5 kg/m^2^), respectively. Among participants, 61.5% were classified as overweight and had no family history of cancer (84%). Additionally, more than 85% of the women were married and had children. Further details on the demographic characteristics of the participants are presented in Table 1. Among the 95 BC samples included in the study, the distribution of subtypes was as follows: Luminal A: 44 (46.3%), Luminal B: 28 (29.5%), TNBC: 8 (8.5%), and HER2+: 15 (15.7%). The stage distribution was also as follows: Stage I: 20 (21.0%), Stage II: 38 (40.4%), Stage III: 30 (31.2%), Stage IV: 7 (7.4%). The tumor grade distribution was as follows: Grade I: 15 (15.8%), Grade II: 56 (58.9%), and Grade III: 24 (25.3%). More details are also presented in Table S1. The subtype distribution in our cohort approximates population‐level BC epidemiology, though TNBC (8.5%) is slightly underrepresented compared to typical reported rates of 10%–15%, and HER2+ (15.7%) is within the expected range of 10%–20%.

**Table 1:** Demographic characteristics of the samples.

No.	Parameter	Category	*N*	(%)
1	Family history	Without BC	80	84.2
		BC	11	11.5
		Other cancers	4	4.3
2	Age (year)	≤50	62	65.2
		>50	33	34.8
		Min–max	20–63	—
		Mean ± SD	44.6 ± 12.3	—
3	Body mass index (kg/m^2^)	≤25	37	38.9
		>25	58	61.1
		Min–max	14.3–42.5	—
		Mean ± SD	26.5 ± 6.2	—
4	Menstruation	Premenopause	54	56.8
		Postmenopause	41	43.2
5	Marital status	Single	11	11.6
		Married	84	88.4
6	Having children	No	13	13.7
		Yes	82	86.3
7	Molecular subtype	Luminal A	44	46.3
		Luminal B	28	29.5
		HER2+	15	15.7
		Triple‐negative (TNBC)	8	8.5
8	Tumor stage	Stage I	20	21.0
		Stage II	38	40.4
		Stage III	30	31.2
		Stage IV	7	7.4
9	Tumor grade	Grade I	15	15.8
		Grade II	56	58.9
		Grade III	24	25.3

### 3.2. Gene Expression and Clinical Features

**Figure 1:**
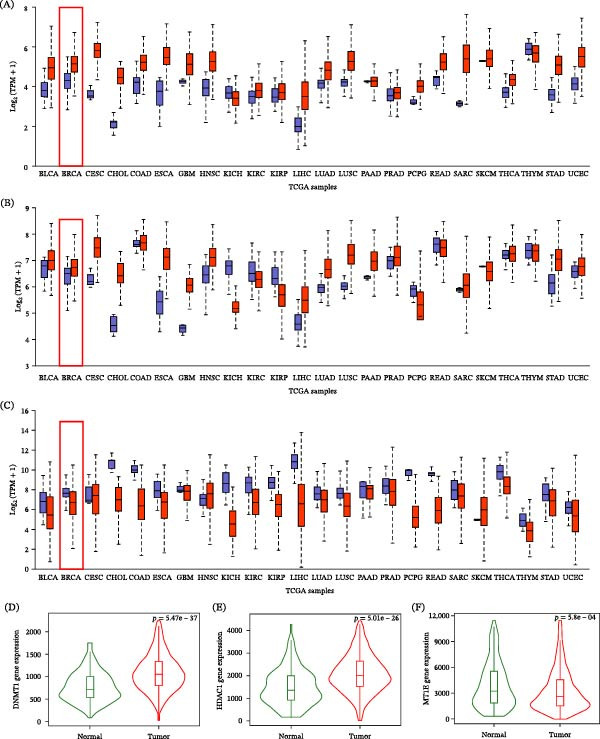
Expressed levels of DNMT1, HDAC1, and MT1E in breast cancer and in pan‐cancers (A–C). The comparison of expression levels of DNMT1, HDAC1, and MT1E in tumor and normal breast cancer (D–F).

Pan‐cancer expression analysis of DNMT1, HDAC1, and MT1E across various tumors is shown in Figure 1A–C. DNMT1 and HDAC1 are upregulated in most tumor types, indicating higher gene expression in cancer cells. In contrast, MT1E is downregulated, suggesting lower expression in tumors. The in silico expression levels of DNMT1, HDAC1, and MT1E in tumor versus normal samples are shown in Figure 1D–F. This TCGA‐based bioinformatics analysis further confirmed that DNMT1 and HDAC1 expression levels were elevated, whereas MT1E expression was reduced. These findings demonstrate consistent directional changes across both in silico and experimental analyses (Figure 1). Statistical comparisons between BC patients and healthy controls were performed, and the detailed results are presented in Tables S2 and S3. After adjusting for age, BMI, tumor subtype, and tumor stage, multivariate logistic regression analysis revealed that MT1E was significantly associated with reduced BC risk (adjusted OR = 0.069, 95% CI: 0.021–0.229, *p*  < 0.001), while DNMT1 (adjusted OR = 2.13, 95% CI: 1.25–4.12, *p*  < 0.05) and HDAC1 (adjusted OR = 1.73, 95% CI: 1.01–2.90, *p*  < 0.05) showed positive associations with increased risk.

**Figure 2:**
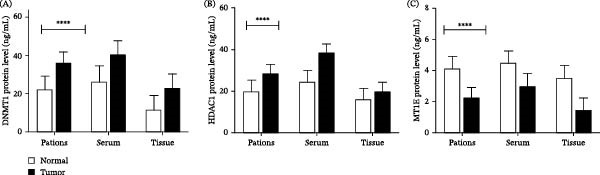
ELISA expression levels of DNMT1, HDAC1, and MT1E proteins in tissue and serum samples (A–C). The asterisks ( ^∗∗∗∗^) represent the significance levels.

**Figure 3:**
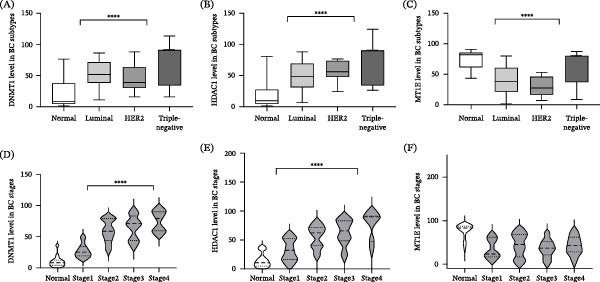
Relationships between DNMT1, HDAC1, and MT1E expression and clinical characteristics. DNMT1, HDAC1, and MT1E expression correlation with tumor subtypes (A–C). DNMT1, HDAC1, and MT1E expression correlation with tumor stage (D–F). The asterisks ( ^∗∗∗∗^) represent the significance levels.

ELISA was used to quantify serum and tissue protein levels expressed by DNMT1, HDAC1, and MT1E, and the results are presented in Figure 2A–C (values of the protein concentration levels expressed by the genes are reported in Table S4). DNMT1 protein levels in all the samples (both tissue and serum) were significantly (*p*‐value < 0.0001) higher than those in controls. Similarly, HDAC1 protein levels in the tissue and serum samples were also significantly (*p*‐value < 0.0001) higher than those in controls. However, MT1E protein levels were significantly (*p*‐value < 0.0001) lower in the BC patient samples. These results are in line with the bioinformatics data (Figure 1), further supporting their relevance in BC. To assess clinical correlations, we examined whether tumor subtypes and stages were associated with DNMT1, HDAC1, and MT1E expression. Figure 3 summarizes these relationships between DNMT1, HDAC1, and MT1E expression and clinical characteristics. Although MT1E was downregulated in BC patients overall compared to healthy controls, within the patient group, the TNBC subtype showed significantly higher MT1E expression compared to luminal and HER2+ subtypes (*p*‐value < 0.0001). This suggests that while MT1E is generally suppressed in BC, its relative level differs across molecular subtypes, with TNBC exhibiting the least suppression. To validate our experimental finding of elevated MT1E in TNBC, we performed an in silico analysis using the TCGA‐BRCA dataset (*n* = 1085 samples) via the UALCAN platform. As shown in Table S5, MT1E mRNA expression was significantly higher in triple‐negative/basal‐like BC (median = 68 transcripts per million) compared to luminal (median = 45) and HER2‐positive (median = 30) subtypes (*p*  < 0.001). This independent external validation confirms that the elevated MT1E expression in TNBC is a reproducible pattern and is not limited to our cohort. Regarding tumor stage, advanced tumor stages showed elevated HDAC1 and DNMT1 expression, whereas MT1E expression was not stage‐dependent.

### 3.3. Identification and Analysis of DEGs

**Figure 4:**
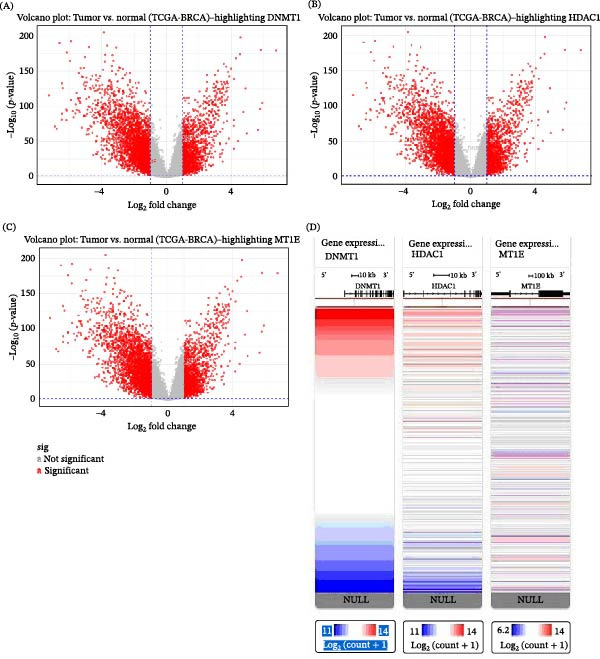
Identification of DEGs in DNMT1, HDAC1 and MT1E genes. The volcano plot showing all the expressed genes from DNMT1, HDAC1 and MT1E (A–C). The heatmap represents the differential expression profiles of DNMT1, HDAC1 and MT1E (|log_2_FC| > 1 and FDR < 0.05) (D).

We used the LinkedOmics database and the UCSC Xena Browser to identify DEGs for DNMT1, HDAC1, and MT1E. For each target gene, samples were dichotomized into high‐ and low‐expression groups based on the median expression level. Genes with |log2FC| > 1 and FDR‐adjusted *p*‐value < 0.05 (Benjamini‐Hochberg correction) were considered significantly differentially expressed. Positively and negatively correlated genes were visualized in a heatmap, and a volcano plot of DEGs was generated to illustrate the overall distribution of significantly altered genes. Positively and negatively correlated genes were visualized in a heatmap, and a volcano plot of DEGs was generated to illustrate the overall distribution of significantly altered genes (Figure 4A–D).

**Figure 5:**
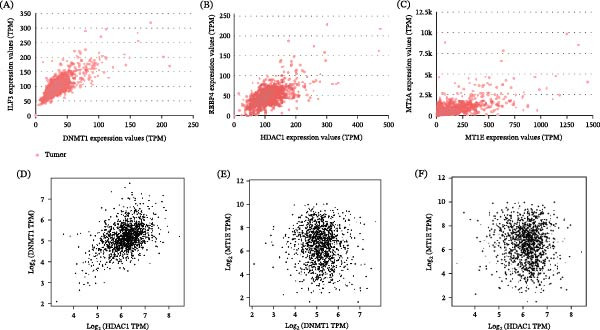
The correlation analysis. The highest correlation partners of DNMT1, HDAC1, and MT1E were identified as ILF3, RBBP4, and MT2A, respectively (A–C). The intercorrelation among DNMT1, HDAC1, and MT1E in BC (D–F).

The volcano plots for DNMT1, HDAC1, and MT1E revealed genes with significant expression changes. This highlights key genes associated with these three targets. Additionally, the heatmap displays genes that were either positively or negatively correlated with DNMT1, HDAC1, and MT1E, illustrating their coexpression patterns, showing which genes increase or decrease together and which exhibit opposing trends. The results of the correlation analysis in BC demonstrated that the genes DNMT1, HDAC1, and MT1E exhibited the highest correlations with ILF3, RBBP4, and MT2A, respectively (Figure 5A–C). Furthermore, the scatter plot illustrating the relationships among DNMT1, HDAC1, and MT1E in the BC samples is shown (Figure 5D–F).

### 3.4. Pathway and Enrichment Analysis

**Figure 6:**
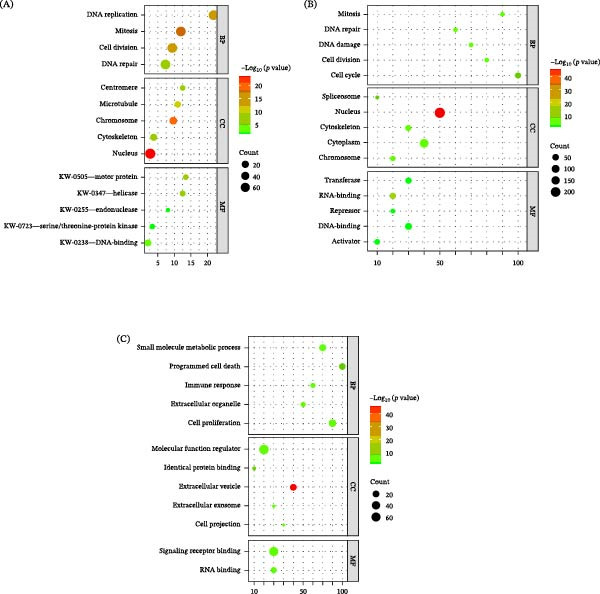
Enrichment analysis of DNMT1, HDAC1, and MT1E genes. Gene ontology (GO) enrichment results for biological process (BP), cellular component (CC), and molecular function (MF) are shown in panels (A–C), respectively.

To identify the pathways and enrichment profiles of the DNMT1, HDAC1, and MT1E genes, DEG enrichment analysis was performed. This generated plots of biological processes, molecular functions, and cellular components, as shown in Figure 6A–C.

GO analysis revealed that these common DEGs were more associated with cell division, differentiation, and nuclear functions. Additionally, KEGG pathway analysis indicated that DNMT1 was linked to cellular senescence and the p53 signaling pathway, whereas HDAC1 was associated with mismatch repair, and MT1E showed a correlation with cytokine–cytokine receptor interaction (Table 2).

**Table 2:** KEGG pathway analysis of differentially expressed genes (*p*‐value < 0.05).

Category	ID	Term	*p*‐Value	Gene
KEGG_PATHWAY	hsa04218	Cellular senescence	0.001	DNMT1
	hsa04115	p53 signaling pathway	0.001	
	hsa04114	Oocyte meiosis	0.019	
	hsa05166	Human T‐cell leukemia virus 1 infection	0.027	
	hsa04914	Progesterone‐mediated oocyte maturation	0.001	
	hsa03410	Base excision repair	0.044	
	hsa03430	Mismatch repair	0.003	HDAC1
	hsa05017	Spinocerebellar ataxia	0.003	
	hsa03015	mRNA surveillance pathway	0.025	
	hsa03440	Homologous recombination	0.033	
	hsa04060	Cytokine–cytokine receptor interaction	0.014	MT1E
	hsa05166	Human T‐cell leukemia virus 1 infection	0.019	
	hsa04062	Chemokine signaling pathway	0.025	
	hsa01100	Metabolic pathways	0.033	

### 3.5. Validation of Genes

**Figure 7:**
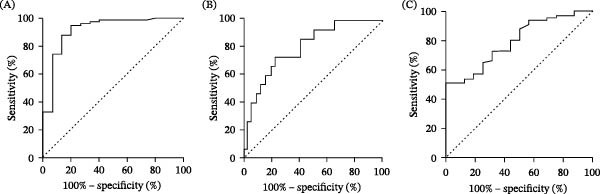
Diagnostic value of the DNMT1 (A), HDAC1 (B), and MT1E (C) genes in identifying normal and breast cancer tissues.

The diagnostic value of DNMT1, HDAC1, and MT1E protein levels was evaluated using ROC analysis (Figure 7A–C and Table S6). The ROC analysis yielded the following results for DNMT1, HDAC1, and MT1E: AUC = 0.916, 0.792, and 0.683; sensitivity = 74.32%, 86.67%, and 50.77%; specificity = 93.33%, 59.46%, and 97.35%, respectively. These findings demonstrate that DNMT1, HDAC1, and MT1E can distinguish BC patients from healthy controls with good accuracy. All ROC analyses were performed with a 95% CI, and *p*  < 0.0001 was considered statistically significant. We also performed an additional in silico ROC analysis using the independent public database TCGA‐BRCA. The analysis was conducted on the same three genes (DNMT1, HDAC1, and MT1E), yielding AUCs of 0.840 for DNMT1, 0.895 for HDAC1, and 0.627 for MT1E (95% CI). These results confirmed the diagnostic potential of all three biomarkers, with AUC values consistent with our original findings. Detailed results are provided in Table S7.

### 3.6. miRNA‐mRNA Network Analysis

**Figure 8:**
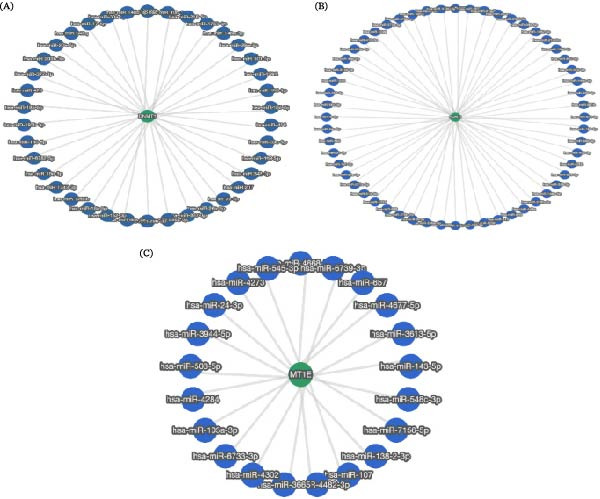
The interaction network between DNMT1 (A), HDAC1 (B), and MT1E (C) and target miRNAs.

miRNA–mRNA interaction analysis was performed using miRTargetLink 2.0, which categorizes interactions as “strongly validated” (supported by reporter assays or Western blot) or “weakly validated” (supported by microarray or sequencing data only). The total number of miRNA interactions for DNMT1 was 114, of which 37 were strongly validated. For HDAC1, a total of 86 interactions were identified, including 10 strongly validated targets. MT1E showed 23 total interactions, all of which were weakly validated, with no strongly validated targets being identified. The top interacting miRNA for each gene based on the interaction score was hsa‐miR‐103a‐3p for DNMT1, hsa‐miR‐34a‐5p for HDAC1, and hsa‐miR‐126‐3p for MT1E (Figure 8A–C).

### 3.7. Drug–Gene Interaction Analysis

Using the DGIdb interaction score database, a total of 98 drugs were associated with the three genes. After ranking based on interaction scores, the top 61 drugs (HDAC1 = 34, DNMT1 = 26, and MT1E = 1) with the highest scores are shown in Table 3.

**Table 3:** Drug–gene interactions.

Interaction score	Interaction types and directionality	Drug	Gene
8.24	N/A	Acetylcysteine	MT1E
4.58	N/A	Zebularine	DNMT1
4.58	N/A	Hexahydrocurcumin	DNMT1
4.58	N/A	Mahanine	DNMT1
4.58	N/A	Chembl2063052	DNMT1
4.58	N/A	Tetrahydrocurcumin	DNMT1
3.33	Inhibitor	Decitabine	DNMT1
2.63	N/A	Tubastatin A	HDAC1
2.29	N/A	Antroquinonol	DNMT1
2.29	N/A	Guadecitabine	DNMT1
2.29	N/A	Palifosfamide	DNMT1
2.29	N/A	Fluorocyclopentenylcytosin	DNMT1
1.53	N/A	Bisdemethoxycurcumin	DNMT1
1.32	N/A	Chembl152133	HDAC1
1.32	N/A	Depudecin	HDAC1
1.32	N/A	Chlamydocin	HDAC1
1.32	N/A	Chembl152543	HDAC1
1.14	N/A	Benzanthrone	DNMT1
1.14	N/A	Azacitidine	DNMT1
0.88	N/A	Spiruchostatin C	HDAC1
0.81	Inhibitor	Mocetinostat	HDAC1
0.76	N/A	Demethoxycurcumin	DNMT1
0.73	Inhibitor	Entinostat	HDAC1
0.71	Inhibitor	Belinostat	HDAC1
0.7	Inhibitor	Panobinostat	HDAC1
0.66	N/A	Obp‐801	HDAC1
0.66	N/A	Nanatinostat	HDAC1
0.66	N/A	Tucidinostat	HDAC1
0.57	Inhibitor	Vorinostat	HDAC1
0.51	N/A	Ifosfamide	DNMT1
0.48	N/A	Trichostatin	HDAC1
0.48	Inhibitor	Romidepsin	HDAC1
0.44	N/A	Domatinostat	HDAC1
0.4	N/A	Psammaplin A	HDAC1
0.38	N/A	Chembl580076	DNMT1
0.36	Inhibitor	Tacedinaline	HDAC1
0.33	N/A	Daphnorin	DNMT1
0.33	N/A	Hydroxyurea	DNMT1
0.33	N/A	Floxuridine	DNMT1
0.33	Inhibitor	Givinostat	HDAC1
0.33	Inhibitor	Dacinostat	HDAC1
0.33	N/A	Chidamide	HDAC1
0.33	Inhibitor	Scriptaid	HDAC1
0.29	N/A	Cephalothin	DNMT1
0.29	N/A	Valproic acid	HDAC1
0.28	Inhibitor	Cudc‐101	HDAC1
0.28	N/A	Phenylbutanoic acid	HDAC1
0.24	N/A	Adriamycin	DNMT1
0.24	N/A	Chembl117487	HDAC1
0.24	N/A	Butanoic acid	HDAC1
0.24	N/A	Butyrylhydroxamic acid	HDAC1
0.24	Inhibitor	Apicidin	HDAC1
0.23	N/A	Largazole	HDAC1
0.22	N/A	Ursolic acid	HDAC1
0.16	N/A	Diethylstilbestrol	DNMT1
0.16	Inhibitor	Depakote	HDAC1
0.11	N/A	Mitoxantrone	DNMT1
0.11	N/A	Pracinostat	HDAC1
0.1	N/A	Curcumin	DNMT1
0.1	N/A	Ethyl‐10	HDAC1
0.03	N/A	Cisplatin	DNMT1

## 4. Discussion

This study highlights the critical role of DNMT1, HDAC1, and MT1E as potential biomarkers in BC, with implications for the diagnosis and therapy. Our findings are in line with emerging evidence that epigenetic dysregulation and MT suppression are key drivers of BC progression, particularly in aggressive subtypes such as TNBC.

MT1E plays a crucial role in maintaining metal homeostasis and adapting to oxidative stress [31]. Its mechanism involves binding zinc and copper ions, which regulate intracellular metal levels and scavenges reactive oxygen species (ROS) [32]. Overexpression of MT1E inhibits apoptosis by suppressing ROS‐mediated DNA damage and p53 activation. Additionally, MT1E inhibits apoptosis by interacting with vital transcription factors such as NF‐κB and P53, protecting cells against apoptosis‐inducing agents [33]. Specifically, MTs facilitate uncontrolled cell proliferation by inactivating P53 through zinc‐ion removal and modulating NF‐κB activity, thereby promoting cell survival [33]. Furthermore, it has been hypothesized that MT1E may contribute to chemotherapy resistance by sequestering metal ions necessary for the efficacy of certain drugs, such as platinum‐based chemotherapies, although direct functional evidence in BC is currently lacking [34]. DNMT1 contributes to the epigenetic silencing of tumor‐suppressor genes through the maintenance of DNA methylation patterns [35]. This enzyme hypermethylates promoter regions, including those of BRCA1 and p16INK4a, and drives the CpG island methylator phenotype, which results in genomic instability and aberrant cell proliferation [36]. Additionally, DNMT1 cooperates with HDAC1 to further repress transcriptionally silenced loci [37]. HDAC1 plays a critical role in chromatin remodeling and transcriptional repression [38]. Mechanistically, it deacetylates histones, such as H3K27, leading to chromatin condensation and subsequent repression of tumor‐suppressor genes like p21 and E‐cadherin [39]. Furthermore, HDAC1 can activate pro‐oncogenic pathways, including Wnt/β‐catenin, through the modulation of nonhistone proteins like STAT3. This enzyme also contributes to EMT and stemness by upregulating SNAIL and TWIST . HDAC1 has multiple roles: for example, it stimulates the Wnt target gene and deacetylates p53 both in vivo and in vitro [40, 41]. Furthermore, HDAC1 is implicated in the DNA damage response, regulating acetylation of histone H3 lysine 56 at DNA damage sites [42]. Overexpression of HDAC1 can reduce p53 and von Hippel‐Lindau protein expression, leading to HIF‐1α overexpression and subsequent angiogenesis [43].

Our data demonstrate that DNMT1 and HDAC1 are significantly overexpressed in BC tissues compared to those in normal controls. These findings reinforce prior studies linking DNA hypermethylation (via DNMT1) and histone deacetylation (via HDAC1) to tumor‐suppressor silencing, hormone resistance, and metastatic progression [44, 45]. Notably, the interaction between DNMT1 and HDAC1 observed in co‐immunoprecipitation experiments in other cancer models raises the possibility of a synergistic epigenetic repression mechanism, but this has not been directly demonstrated in our system. In addition, genomic modifications involving DNMT‐related enzymes are closely linked to clinicopathological characteristics in cancer. Histone posttranslational modifications, which occur in either the N‐terminal tails or core domains of histones, play a pivotal role in modulating the chromatin structure and function [7, 46]. These modifications are further regulated by the opposing actions of histone acetyltransferases and HDACs, which dynamically alter chromatin accessibility through acetylation and deacetylation [47]. Notably, transcriptionally silent genes are typically associated with hypoacetylated chromatin, whereas hyperacetylated chromatin marks actively transcribed regions [48]. Supporting the therapeutic relevance of these mechanisms, studies show that HDAC1 knockdown via siRNA suppresses the proliferation and survival of mammalian carcinoma cells, suggesting that HDAC1 is essential for tumor growth and could serve as a promising therapeutic target in BC [49].

We measured serum and tissue concentrations of three proteins (DNMT1, HDAC1, and MT1E) in female BC patients to evaluate potential biomarkers and assess their efficacy in the diagnosis of BC patients using ROC analysis. ROC analysis yielded AUC values of 0.916 for DNMT1, 0.792 for HDAC1, and 0.683 for MT1E. ROC analysis using an independent public dataset (TCGA) confirmed our experimental findings. Compared to normal tissues, BC patients showed significantly elevated DNMT1 and HDAC1 levels but reduced MT1E concentrations in both serum and tissue samples. These experimental findings were supported by complementary in silico analyses.

Pan‐cancer expression profiling revealed consistent upregulation of DNMT1 and HDAC1 coupled with downregulation of MT1E transcription across multiple tumor types. This concordance between our protein‐level measurements in BC and transcriptional patterns observed in various cancers suggests robust cross‐validation of these biomarkers. Our results align with established literature, demonstrating that DNMT1 upregulation at mRNA, protein, and functional levels promotes carcinogenesis [50, 51]. Notably, previous studies have documented particularly pronounced DNMT1 overexpression in estrogen receptor (ER)‐negative BC cell lines at both transcriptional and translational levels [52], with ~3‐fold higher expression observed across various malignancies, including BC [53].

HDAC1 overexpression has been strongly correlated with human tumorigenesis [54] and is observed in ~33% of BC cases [55]. Clinical evidence demonstrates significantly elevated HDAC1 mRNA levels in invasive breast carcinoma patients [15], with its overexpression linked to both gene silencing and reduced ER activity in BC [56]. The established association between HDAC1 and BC metastasis/invasiveness highlights its diverse functional roles across different BC subtypes [56, 57].

Parallel to these findings, MT1E downregulation has emerged as a potential biomarker in prostate cancer [58] and hepatocellular carcinoma [59] while also being implicated in glioma cell motility [60]. Immunohistochemical studies suggest that MT proteins may serve as valuable prognostic markers for invasive breast carcinomas [61, 62], with numerous investigations correlating MT expression with key clinicopathological parameters including histological grade [63, 64] and hormone receptor status [65, 66]. In gastric cancer, MT expression demonstrates significant prognostic value, while in mammary gland cancers, tumor‐promoted MT activity appears to involve zinc‐mediated physiological interactions [67]. Animal studies further support these observations by identifying critical regulatory elements in MT gene promoters [67].

The oncogenic effects of HDAC1 may be amplified through its functional collaboration with DNA methylation mechanisms. Current models suggest that coordinated DNA methylation and histone deacetylation create a repressive chromatin state that silences gene expression [68].

One of the important aspects of our study is that we have shown strong correlations between the expression levels of these genes and certain clinicopathological features, emphasizing their potential clinical relevance. We found that the tumor expression of DNMT1, HDAC1, and MT1E correlated with clinical characteristics and was higher in the TNBC molecular subtype (*p*‐value < 0.0001). Recent studies highlight the potential key role of HDAC1 in TNBC [69]. One novel finding is that MT1E, while generally downregulated in BC, shows a significantly higher expression in TNBC compared to luminal and HER2+ subtypes. This was independently validated using the TCGA‐BRCA data (Table S5). This finding may appear counterintuitive given that TNBC is the most aggressive subtype. However, this observation remains hypothetical and requires functional validation. Due to the limited TNBC sample size, these findings should be interpreted cautiously until validated in larger cohorts. Several hypothetical mechanisms may explain this pattern, but none have been experimentally tested in this study: First, it is speculative that MT1E could be upregulated as an adaptive response to high oxidative stress in TNBC, but this hypothesis requires direct experimental testing. Second, MT1E has been implicated in chemoresistance to platinum‐based agents. Third, relative hypomethylation of the MT1E promoter in TNBC could partially restore its transcription. These potential mechanisms require direct investigation in future studies. The results also demonstrated that advanced stage tumors exhibited higher expression of HDAC1 and DNMT1 compared to lower stage tumors. These findings contribute to the growing body of evidence that epigenetic modifications, such as DNA methylation and histone acetylation, play crucial roles in tumor initiation and progression. In addition, GO analysis revealed that these common DEGs were significantly enriched in cell division and differentiation processes. Similarly, Chen et al. [70] reported that DEGs were strongly associated with cell cycle progression and proliferation in BC. This demonstrates how GO analysis serves as a key example of using bioinformatics tools to interpret gene‐expression data.

In this study, we identified the differential expression of three genes, DNMT1, HDAC1, and MT1E, and analyzed their interactions with targeting miRNAs. This approach integrates experimental gene expression data with in silico miRNA target prediction. The results revealed 37 strongly validated target genes for DNMT1, 10 for HDAC1, and 23 weakly validated targets for MT1E. The top interacting miRNAs included hsa‐miR‐103a‐3p (targeting DNMT1), hsa‐miR‐34a‐5p (HDAC1), and hsa‐miR‐126‐3p (MT1E). KEGG pathway analysis linked DNMT1 to cellular senescence and the p53 signaling pathway, HDAC1 to mismatch repair, and MT1E to cytokine–cytokine receptor interactions, providing a bioinformatics‐supported understanding of their functional roles. The correlation analysis in BC demonstrated that the genes DNMT1, HDAC1, and MT1E exhibited the highest correlations with ILF3, RBBP4, and MT2A, respectively. ROC analysis further supported the diagnostic potential of these genes, demonstrating excellent discriminatory power between BC and adjacent tissues, which was validated by both experimental and in silico data.

While this study provides compelling initial evidence for the diagnostic potential of the three‐gene panel, several limitations should be considered. First, while the current sample size provided sufficient statistical power for preliminary analyses, future studies should incorporate larger, multicenter, and ethnically diverse cohorts to enhance the generalization and clinical validity of these biomarkers. Second, the cross‐sectional design of this study inherently limits our ability to infer causal relationships or track dynamic changes throughout disease progression; therefore, longitudinal studies are recommended to address this gap. Third, and most importantly, direct functional validation (e.g., siRNA knockdown, CRISPR knockout, or cell proliferation/migration assays in TNBC cell lines) was not performed. Consequently, our findings support biomarker association rather than mechanistic causation. Such experiments, including treatment with DNMT or HDAC inhibitors, are planned for future studies. Furthermore, although molecular correlations (e.g., gene‐to‐gene correlations or miRNA–mRNA interactions) were calculated in silico, the correlation between the expression levels of mRNA and the corresponding protein levels of MT1E, DNMT1, and HDAC1 was not evaluated, which prevents confirmation of the link between transcriptional changes and protein abundance. Finally, comparison with established biomarkers such as CA15−3 and combined biomarker models was not performed and requires future investigation.

## 5. Conclusion

In conclusion, DNMT1, HDAC1, and MT1E are differentially expressed in BC and correlate with clinical characteristics, particularly in the TNBC molecular subtype. The expression levels of DNMT1 and HDAC1 are associated with the tumor stage, with the highest expression observed in advanced‐stage tumors. Serum and tissue expression analyses confirm that DNMT1, HDAC1, and MT1E are potential complementary biomarkers for BC diagnosis. However, these findings are preliminary, and functional studies are required before clinical translation.

## Author Contributions

**Sedigheh Akhtartavan**: conceptualization (supporting), data curation (lead), formal analysis (supporting), investigation (supporting), methodology (supporting), visualization (lead), writing – original draft (lead). **Hamid Madanchi and Ahmad Abdullahi**: project administration (supporting), investigation (supporting), writing – original draft (supporting). **Raheb Ghorbani and Abolfazl Khalafi-Nezhad**: formal analysis (supporting), methodology (lead), investigation (supporting). **Fahimeh Shamsi**: conceptualization (lead), resources (lead), supervision (lead), writing – review and editing (supporting), validation (lead). **Hossein Heli**: conceptualization (supporting), formal analysis (supporting), writing – review and editing (supporting).

## Funding

This research was a part of Sedigheh Akhtartavan’s PhD thesis supported by Semnan University of Medical Sciences, Semnan, Iran.

## Disclosure

All authors contributed to the article and approved the submitted version.

## Ethics Statement

The study was conducted in accordance with the principles of the Declaration of Helsinki and approved by the Local Ethics and Scientific Committee of the Faculty of Medicine, Semnan University of Medical Sciences (IR.SEMUMS.REC.1401.314). Written informed consent was obtained from all participants prior to their involvement in the study.

## Conflicts of Interest

The authors declare no conflicts of interest.

## Supporting Information

Additional supporting information can be found online in the Supporting Information section.

## Supporting information


**Supporting Information** The Supporting Information for this article includes; Table S1: The Demographic characteristics of samples. Tables S2 and S3: The Student’s *t*‐test of MT1E, DNMT1, and HDAC1 for sample comparisons. Table S4: The values of the protein concentration levels expressed by the genes. Table S5: Expression of MT1E across BC subtypes (TCGA‐BRCA). Table S6: The diagnostic value of DNMT1, HDAC1, and MT1E protein levels. Table S7: In silico ROC analysis of DNMT1, HDAC1, and MT1E genes in the TCGA‐BRCA database.

## Data Availability

The original contributions presented in the study are included in the article/Supporting Information. Further inquiries can be directed to the corresponding author.
