# Supplementary material for: Expression Levels of DNMT1, HDAC1, and MT1E Genes as Diagnostic Biomarkers for Breast Cancer Clinicopathological Features: In Vitro and In Silico Analyses
Source: Anal Cell Pathol (Amst). 2026 Jul 21;2026:9606190. doi: 10.1155/ancp/9606190 (PMC13386243; doi:10.1155/ancp/9606190)
Supplement: Supplementary file 1 — Supporting Information The Supporting Information for this article includes; Table S1: The Demographic characteristics of samples. Tables S2 and S3: The Student’s t‐test of MT1E, DNMT1, and HDAC1 for sample comparisons. Table S4: The values of the protein concentration levels expressed by the genes. Table S5: Expression of MT1E across BC subtypes (TCGA‐BRCA). Table S6: The diagnostic value of DNMT1, HDAC1, and MT1E protein levels. Table S7: In silico ROC analysis of DNMT1, HDAC1, and MT1E genes in the TCGA‐BRCA database. [file ANCP-2026-9606190-s001.zip › S2.doc]

(A)

(B)

(C)

Supplementary material S2: Standard curve of DNMT1 (A), HDAC1 (B), and MT1E(C).
