# Supplementary material for: Expression Levels of DNMT1, HDAC1, and MT1E Genes as Diagnostic Biomarkers for Breast Cancer Clinicopathological Features: In Vitro and In Silico Analyses
Source: Anal Cell Pathol (Amst). 2026 Jul 21;2026:9606190. doi: 10.1155/ancp/9606190 (PMC13386243; doi:10.1155/ancp/9606190)
Supplement: Supplementary file 1 — Supporting Information The Supporting Information for this article includes; Table S1: The Demographic characteristics of samples. Tables S2 and S3: The Student’s t‐test of MT1E, DNMT1, and HDAC1 for sample comparisons. Table S4: The values of the protein concentration levels expressed by the genes. Table S5: Expression of MT1E across BC subtypes (TCGA‐BRCA). Table S6: The diagnostic value of DNMT1, HDAC1, and MT1E protein levels. Table S7: In silico ROC analysis of DNMT1, HDAC1, and MT1E genes in the TCGA‐BRCA database. [file ANCP-2026-9606190-s001.zip › S3.doc]

Supplementary material S3: The student’s t-test of DNMT1, HDAC1, and MT1E.

|  | Statistical Parameter | DNMT1 | HDAC1 | MT1E |
| --- | --- | --- | --- | --- |
| Variance homogeneity (F-test) | p-value (F-test) | 0.14330 | 0.85700 | 0.09500 |
| t-test Results | t-value | 4.713 | 7.162 | 20.32 |
|  | df | 86 | 87 | 86 |
|  | p-value (t-test) | <0.0001 | <0.0001 | <0.0001 |
|  | Mean expression (normal) | 22.56 | 20.00 | 5.470 |
|  | Mean expression (tumor) | 40.61 | 29.50 | 2.63 |
|  | Sample size (N) | 50 (control), 95 (BC) | 50 (control), 95 (BC) | 50 (control), 95 (BC) |
