# Supplementary material for: Expression Levels of DNMT1, HDAC1, and MT1E Genes as Diagnostic Biomarkers for Breast Cancer Clinicopathological Features: In Vitro and In Silico Analyses
Source: Anal Cell Pathol (Amst). 2026 Jul 21;2026:9606190. doi: 10.1155/ancp/9606190 (PMC13386243; doi:10.1155/ancp/9606190)
Supplement: Supplementary file 1 — Supporting Information The Supporting Information for this article includes; Table S1: The Demographic characteristics of samples. Tables S2 and S3: The Student’s t‐test of MT1E, DNMT1, and HDAC1 for sample comparisons. Table S4: The values of the protein concentration levels expressed by the genes. Table S5: Expression of MT1E across BC subtypes (TCGA‐BRCA). Table S6: The diagnostic value of DNMT1, HDAC1, and MT1E protein levels. Table S7: In silico ROC analysis of DNMT1, HDAC1, and MT1E genes in the TCGA‐BRCA database. [file ANCP-2026-9606190-s001.zip › S4.doc]

Supplementary material S4: ELISA assay results: Protein expression levels of MT1E, DNMT1, and HDAC1 in serum and tissue samples.

| Gene | Sample type | Protein concentration-BC / ng/mL | Control / ng/mL |
| --- | --- | --- | --- |
| DNMT1 | Serum | 40.6 | 22.5 |
|  | Tissue | 22.9 | 11.9 |
| HDAC1 | Serum | 39.6 | 22.5 |
|  | Tissue | 19.9 | 17.1 |
| MT1E | Serum | 2.63 | 5.1 |
|  | Tissue | 1.8 | 3.9 |
