# Supplementary material for: Expression Levels of DNMT1, HDAC1, and MT1E Genes as Diagnostic Biomarkers for Breast Cancer Clinicopathological Features: In Vitro and In Silico Analyses
Source: Anal Cell Pathol (Amst). 2026 Jul 21;2026:9606190. doi: 10.1155/ancp/9606190 (PMC13386243; doi:10.1155/ancp/9606190)
Supplement: Supplementary file 1 — Supporting Information The Supporting Information for this article includes; Table S1: The Demographic characteristics of samples. Tables S2 and S3: The Student’s t‐test of MT1E, DNMT1, and HDAC1 for sample comparisons. Table S4: The values of the protein concentration levels expressed by the genes. Table S5: Expression of MT1E across BC subtypes (TCGA‐BRCA). Table S6: The diagnostic value of DNMT1, HDAC1, and MT1E protein levels. Table S7: In silico ROC analysis of DNMT1, HDAC1, and MT1E genes in the TCGA‐BRCA database. [file ANCP-2026-9606190-s001.zip › S6.doc]

Supplementary material S6: Sensitivity, specificity and AUC of the potential predictors.

| Gene | Cut-off | Sensitivity | Specificity | p-value | 95% confidence interval | AUC |
| --- | --- | --- | --- | --- | --- | --- |
| DNMT1 | > 36.83 | 74.32 | 93.33 | 0.0001 | 0.8257 to 1.000 | 0.9167 |
| HDAC1 | > 32.86 | 86.67 | 59.46 | 0.0001 | 0.6984 to 0.9250 | 0.7928 |
| MT1E | > 2.38 | 50.77 | 97.35 | 0.0001 | 0.5835 to 0.9021 | 0.6835 |
